# Role of the *Wnt *receptor Frizzled-1 in presynaptic differentiation and function

**DOI:** 10.1186/1749-8104-4-41

**Published:** 2009-11-02

**Authors:** Lorena Varela-Nallar, Catalina P Grabowski, Iván E Alfaro, Alejandra R Alvarez, Nibaldo C Inestrosa

**Affiliations:** 1 Centro de Envejecimiento y Regeneración (CARE), Centro de Regulación Celular y Patología "Joaquín V Luco" (CRCP) and MIFAB, Chile; 2 Laboratorio de Señalización Celular, Departamento de Biología Celular y Molecular, Facultad de Ciencias Biológicas, Pontificia Universidad Católica de Chile, Santiago, Chile

## Abstract

**Background:**

The *Wnt *signaling pathway regulates several fundamental developmental processes and recently has been shown to be involved in different aspects of synaptic differentiation and plasticity. Some *Wnt *signaling components are localized at central synapses, and it is thus possible that this pathway could be activated at the synapse.

**Results:**

We examined the distribution of the *Wnt *receptor Frizzled-1 in cultured hippocampal neurons and determined that this receptor is located at synaptic contacts co-localizing with presynaptic proteins. Frizzled-1 was found in functional synapses detected with FM1-43 staining and in synaptic terminals from adult rat brain. Interestingly, overexpression of Frizzled-1 increased the number of clusters of Bassoon, a component of the active zone, while treatment with the extracellular cysteine-rich domain (CRD) of Frizzled-1 decreased Bassoon clustering, suggesting a role for this receptor in presynaptic differentiation. Consistent with this, treatment with the Frizzled-1 ligand *Wnt-3a *induced presynaptic protein clustering and increased functional presynaptic recycling sites, and these effects were prevented by co-treatment with the CRD of Frizzled-1. Moreover, in synaptically mature neurons *Wnt-3a *was able to modulate the kinetics of neurotransmitter release.

**Conclusion:**

Our results indicate that the activation of the *Wnt *pathway through Frizzled-1 occurs at the presynaptic level, and suggest that the synaptic effects of the *Wnt *signaling pathway could be modulated by local activation through synaptic Frizzled receptors.

## Background

The *Wnt *signaling pathway plays a crucial role during development, regulating specification of cell fate, cell proliferation, migration and morphogenesis [1]. *Wnt *signaling is activated by the interaction of *Wnt *ligands with members of the Frizzled (Fz) family of seven-transmembrane cell surface receptors. Three different *Wnt *pathways have been described downstream of Fz receptors: the canonical *Wnt*/β-catenin pathway; and the non-canonical pathways involving intracellular signaling by Ca^2+ ^or the c-Jun-N-terminal kinase (JNK) cascade [1,2]. In the canonical *Wnt*/β-catenin signaling pathway, *Wnt *ligands interact with Fz receptors and their co-receptor LRP5/6 and signal through Dishevelled to inhibit the kinase activity of glycogen synthase kinase-3β in a protein degradation complex containing Axin and adenomatous polyposis coli (APC) protein. When *Wnt *signaling is inactive, β-catenin is phosphorylated by glycogen synthase kinase-3β and thus rapidly degraded via the proteasome pathway. When cells receive *Wnt *signals, the degradation pathway is inhibited, and β-catenin consequently accumulates in the cytoplasm and is translocated to the nucleus where it binds the TCF/LEF family of transcription factors to regulate the expression of *Wnt *target genes [1].

Fz receptors have an extracellular amnio-terminal region that contains a cysteine-rich domain (CRD) consisting of 120 to 125 residues with 10 conserved cysteines that is necessary for the binding of *Wnt *molecules [3,4]. In mammals, 19 different *Wnts *are known, and 10 Fz proteins have been identified as *Wnt *receptors. In addition to Fz, other *Wnt *receptors have been described more recently [2,5], and it has been shown that a single *Wnt *ligand can signal through different pathways depending on receptor context [6], increasing the complexity of the *Wnt *signaling cascade.

In the past decade, it has been well established that *Wnt *signaling plays a key role in diverse aspects of neuronal connectivity by regulating axon guidance and remodeling [7,8], dendritic development [9], and synapse formation [8,10,11]. Additionally, intracellular modulators of the *Wnt *pathway enhanced excitatory transmission in adult hippocampal preparations, acting predominantly via a presynaptic mechanism to increase neurotransmitter release [12], and *Wnt-7a *was shown to induce recycling and exocytosis of synaptic vesicles in cultured hippocampal neurons and enhance synaptic transmission in adult hippocampal slices [13]. Furthermore, *Wnt-3a *is released from synapses in an activity-dependent manner, and the secreted *Wnt *and the consequent activation of *Wnt *signaling facilitates long-term potentiation, suggesting that *Wnt *signaling plays a role in regulating synaptic plasticity [14]. *Wnt-3a *was also shown to induce the recycling of synaptic vesicles in cultured hippocampal neurons [13].

In the present work, we have studied the distribution of the canonical *Wnt *receptor Fz1 in neurons, and the potential role of this receptor in synapse structure and function. We determined that, in cultured hippocampal neurons, Fz1 clusters are localized in synapses co-localizing with presynaptic markers in close apposition to the postsynaptic protein PSD-95. In addition, Fz1 was observed in functional synapses detected with FM1-43 staining and in synaptic terminals from adult rat brain. Overexpression of Fz1 increased the number of Bassoon clusters, and treatment with the Fz1 ligand *Wnt-3a *induced presynaptic protein clustering and modulated the kinetics of synaptic vesicle release, suggesting that the activation of *Wnt *signaling through Fz1 modulates presynaptic differentiation and function.

## Results

### Presynaptic distribution of the Fz1 receptor in hippocampal neurons

**Figure 1:**
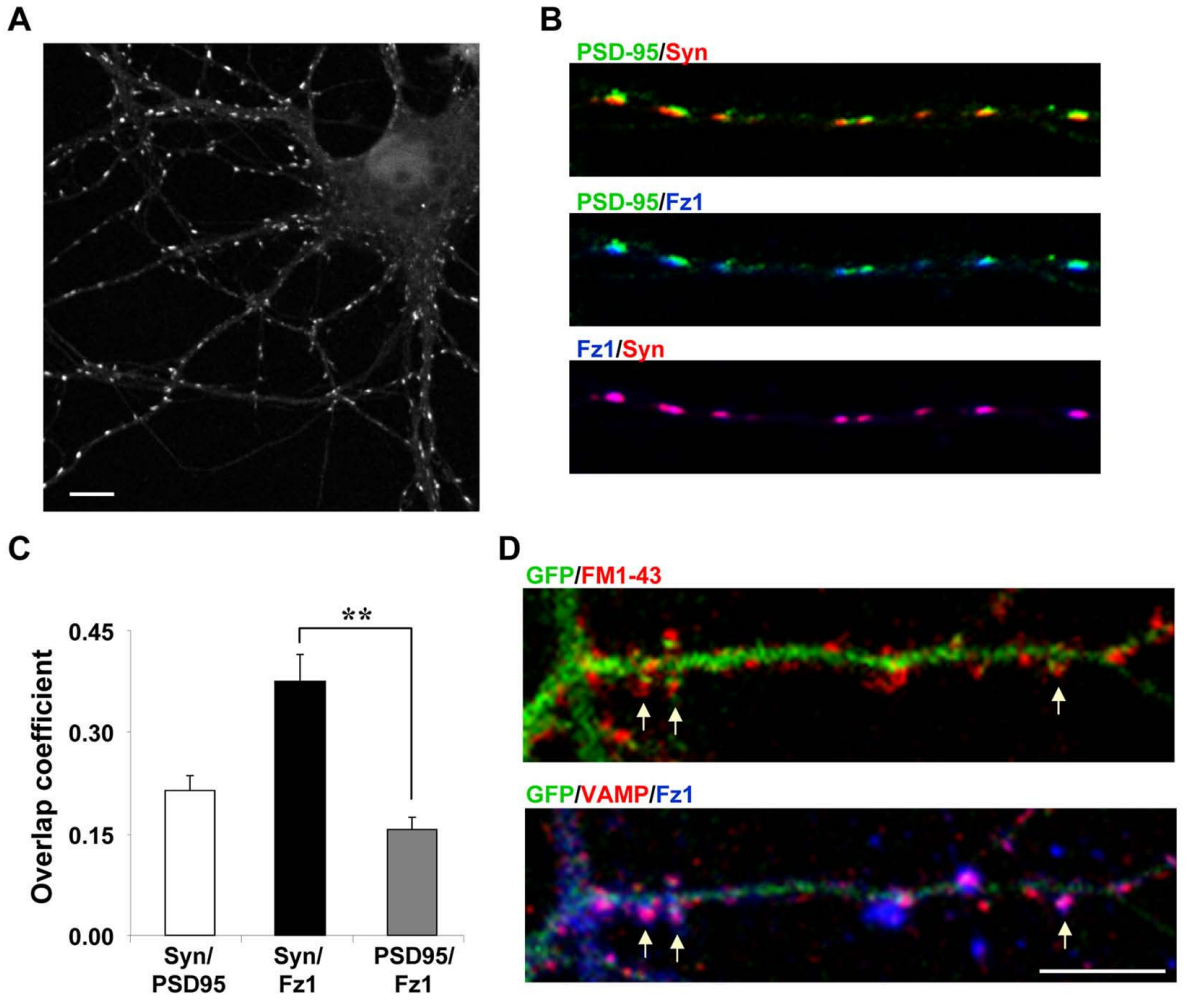
**Fz1 is located at presynaptic sites in hippocampal neurons**. **(A) **Immunodetection of Fz1 in hippocampal neurons maintained for 14 days in culture shows a clustered distribution of the receptor (scale bar = 10 μm). **(B) **Representative merged images with the postsynaptic marker PSD-95 and the presynaptic protein synapsin I (Syn) indicate that Fz1 clusters are located presynaptically in close apposition to PSD-95 puncta. **(C) **Overlap coefficient according to Manders *et al*. [17] indicates an overlap of Fz1 and Syn signals. **(D) **Synaptic vesicles were labeled by FM1-43 uptake in hippocampal neurons transfected with GFP. After fixation and permeabilization, cells were inmunostained with anti-VAMP and anti-Fz1 and the same GFP-positive neuron was analyzed by confocal microscopy. As observed, Fz1 is present in most of the presynaptic terminals stained with FM1-43 and VAMP (arrows; scale bar = 10 μm). Error bars indicate standard error of the mean. **P < 0.01.

In order to better understand the mechanisms involved in the synaptic effects of the *Wnt *pathway, we studied the distribution of the Fz1 receptor, which has been well studied and has been related to the *Wnt*/β-catenin pathway [15,16]. The distribution of Fz1 was analyzed by immunofluorescence in cultured hippocampal neurons at 14 days *in vitro *(DIV). This receptor shows a punctate distribution in neuronal processes and a low staining in the cell body (Figure 1A). To test whether Fz1 is present at synaptic sites in cultured neurons, we examined the co-localization of Fz1 with the presynaptic marker synapsin I (Syn) and the postsynaptic density scaffold protein PSD-95. Fz1 clusters are in close apposition to PSD-95 puncta and seem to co-localize with Syn (Figure 1B). This observation is supported by the overlap coefficient according to Manders *et al*. [17], which represents the number of co-localized pixels expressed as a fraction of the total number of pixels within each channel (Syn/PSD-95, 0.214 ± 0.023; Syn/Fz1, 0.376 ± 0.039; PSD-95/Fz1, 0.157 ± 0.016; Figure 1C). In addition, another approach was used to establish the presynaptic distribution of Fz1. In hippocampal neurons transfected with green fluorescent protein (GFP), synaptic vesicles were labeled by activity-dependent uptake of the fluorescent dye FM1-43 and analyzed by confocal microscopy (Figure 1D, upper panel). Then, cells were fixed and permeabilized and the presynaptic protein VAMP and Fz1 were detected by immunofluorescence. The same GFP-positive neuron was analyzed by confocal microscopy (Figure 1D, lower panel). Fz1 was present in most of the presynaptic terminals stained with FM1-43 and VAMP (Figure 1D, arrows). Altogether, these data strongly suggest that Fz1 is located presynaptically.

### Localization of Fz1 in functional synapses, during development and in synaptosomal fractions of adult rat brain

**Figure 2:**
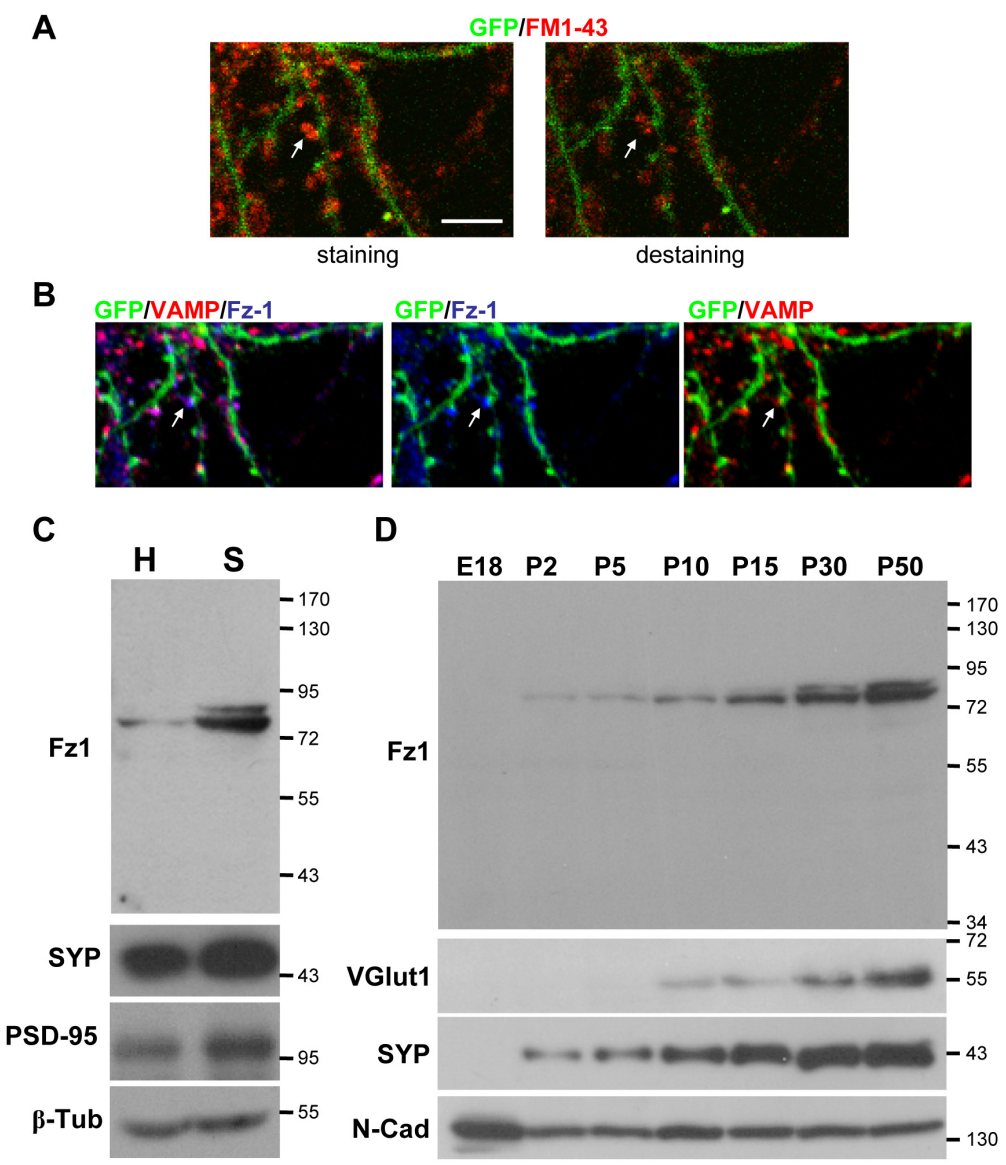
**Expression of Fz1 in functional synapses, synaptosomal fractions from adult brain and during hippocampal development**. **(A) **In hippocampal neurons transfected with GFP, synaptic vesicles were labeled by activity-dependent uptake of FM1-43 and subsequently destained by depolarization with 90 mM KCl to identify functional synapses (arrow, scale bar = 5 μm). **(B) **After fixation and permeabilization, neurons were inmunostained with anti-VAMP and anti-Fz1 antibodies and the same GFP-positive neuron was analyzed by confocal microscopy. Fz-1 is present in most of the synaptic terminals that were destained by depolarization and display immunoreactivity for VAMP (arrow). **(C) **Analysis of the total homogenized (H) and synaptosomal fractions (S) obtained from adult rat brains shows that Fz1 is enriched in the synaptosomal fraction. PSD-95 and synatophysin (SYP) were used as synaptic markers. **(D) **Immunoblots of proteins in the developing hippocampus from embryonic day 18 (E18) and postnatal days 2 (P2) to P50. The same amount of protein was applied to all lanes. Molecular weight standards are indicated at the right (kDa). β-Tub, β-tubulin; N-Cad, N-cadherin; VGlut1, vesicular glutamate transporter 1.

To determine whether the Fz1-positive synaptic sites were functional synapses, synaptic vesicles of hippocampal neurons transfected with GFP were labeled by activity-dependent uptake of FM1-43 (Figure 2A, left panel) and then destained by depolarization with 90 mM KCl (Figure 2A, right panel). After fixation and permeabilization, neurons were inmunostained with anti-VAMP and anti-Fz1 antibodies and the same GFP-positive neuron was analyzed by confocal microscopy. Fz1 is present in most of the presynaptic terminals that were stained with FM1-43 and destained by depolarization, and that are also positive for VAMP (Figure 2B, arrow), indicating that this receptor is present in functional synapses at the presynaptic site.

In addition, we analyzed the presence of Fz1 in synaptosomal fractions isolated from adult brains and observed that, in agreement with the distribution observed by immunofluorescence, this receptor is enriched in this fraction (Figure 2C). The specificity of the synaptosomal fraction was confirmed by the presence of the synaptic proteins PSD-95 and synaptophysin (SYP).

In an attempt to find out whether Fz1 participates in synapse formation, we studied the expression of Fz1 in the developing hippocampus, and observed that this receptor is undetectable before birth, and increases from a small amount at P2 to high levels in adult brain, as was also observed for the synaptic proteins vesicular glutamate transporter 1 (VGlut1) and SYP (Figure 2D) [18]. N-cadherin was used as a loading control since it is known to be present at all developmental stages [19].

**Figure 3:**
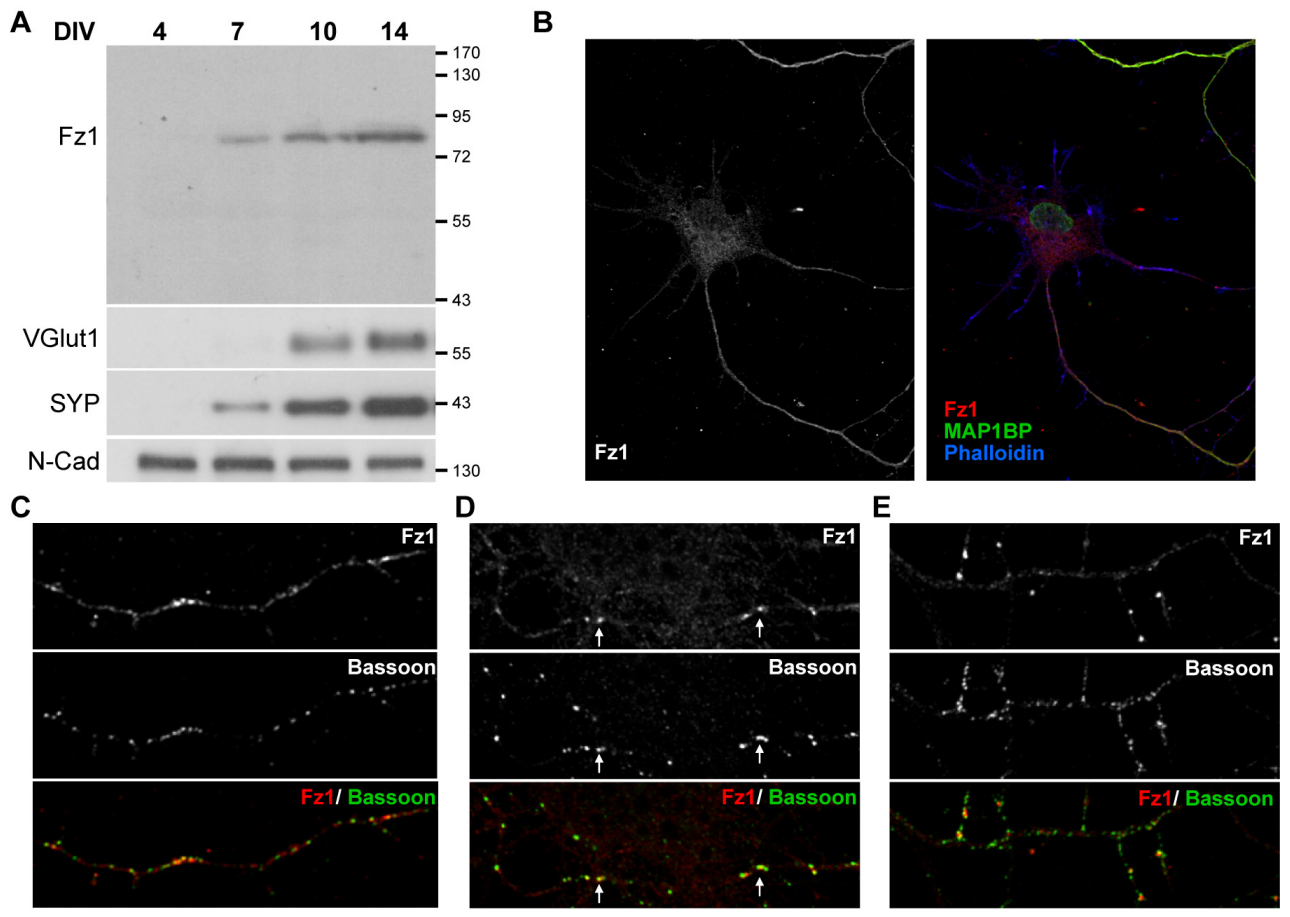
**Expression of Fz1 in hippocampal neurons during development**. **(A) **Immunoblots of protein extracts from cultured hippocampal neurons during differentiation from 4 to 14 DIV. The same amount of protein was applied to all lanes. Molecular weight standards are indicated at the right (kDa). N-Cad, N-cadherin. **(B) **Immunodetection of Fz1 and phosphorylated MAP1B in cultured hippocampal neurons at 4 DIV. **(C-E) **Immunodetection of Fz1 and Bassoon (scale bar = 10 μm). (C) On DIV 7, Fz1 is observed in isolated axons as fine puncta. (D) On DIV 10, larger clusters of Fz1 co-localize with Bassoon puncta in axons contacting other neurons (arrows), but not in isolated axons (E).

We also analyzed the temporal appearance of Fz1 in differentiating hippocampal neurons, and observed an increase of Fz1 during development concomitant with the increase of VGlut1 and SYP levels (Figure 3A). The spatial appearance of Fz1 was also analyzed by immunofluorescence. At early stages of differentiation (4 DIV), Fz1 distributes mostly at the axon as determined by double labeling with the axonal marker phosphorylated MAP1B [20] (Figure 3B), supporting the presynaptic distribution of this receptor. By 7 DIV, Fz1 is observed in isolated axons as fine puncta that mostly do not co-localize with Bassoon puncta (Figure 3C), suggesting that Fz1 is not associated with presynaptic proteins during the initial stages of synaptic assembly. Bassoon is a presynaptic cytomatrix protein recruited early during synapse formation [21]. By 10 DIV, larger clusters of Fz1 are observed and it is possible to find co-localization of Fz1 with Bassoon mainly in axons contacting other neurons (Figure 3D, arrows), but not in isolated axons (Figure 3E). The number of Fz1 clusters increases steadily during neuronal maturation, suggesting an association of Fz1 with synapse formation.

### Fz1 modulates presynaptic protein clustering

**Figure 4:**
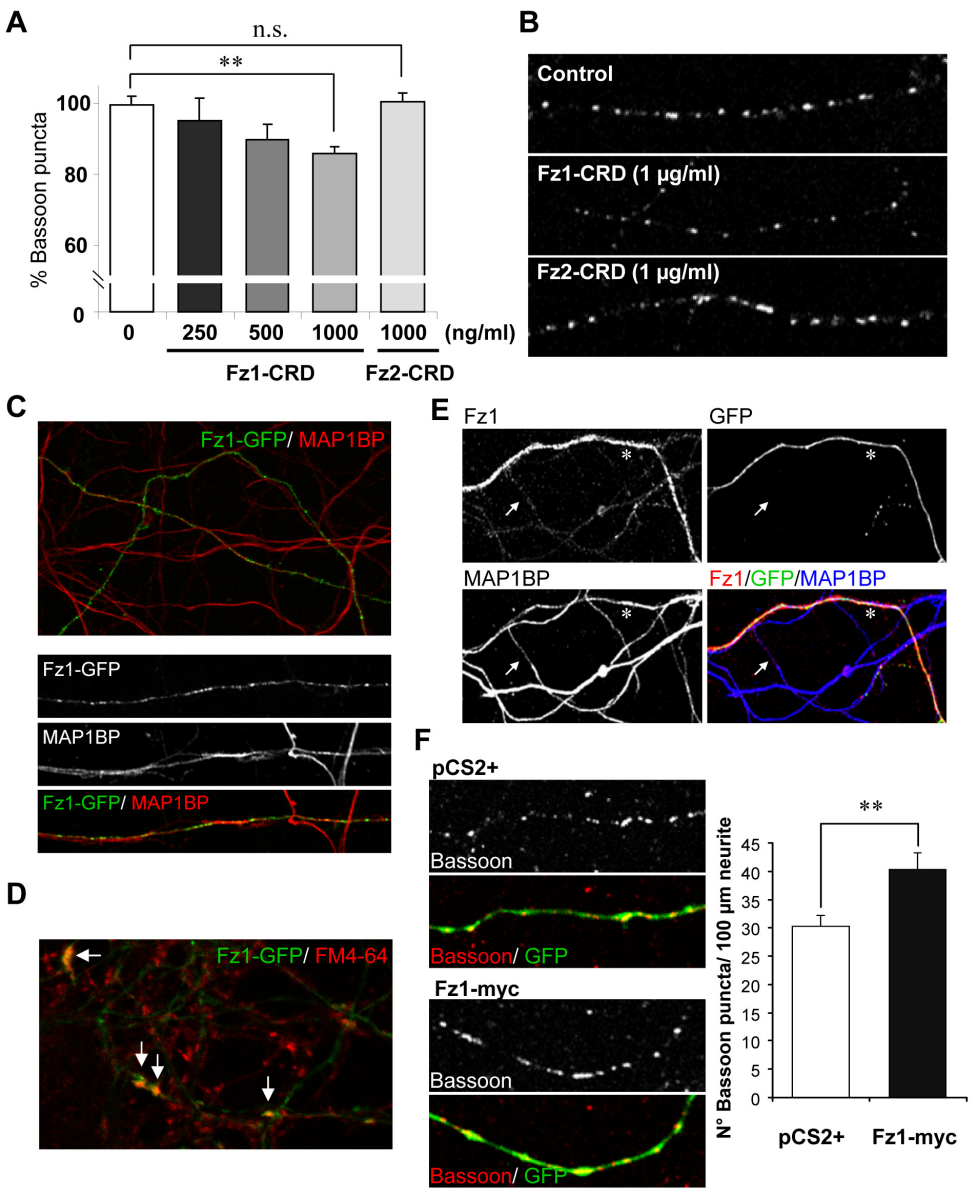
**Fz1 overexpression induces presynaptic protein clustering**. **(A) **The number of Bassoon clusters per neurite length was evaluated in hippocampal neurons (9 DIV) treated for 24 h with different concentrations of the soluble extracellular Fz1 CRD or 1,000 ng/ml Fz2-CRD. **(B) **Representative images of control neurons and neurons treated with 1 μg/ml Fz1-CRD or Fz2-CRD for 24 h. **(C) **Hippocampal neurons were transfected with Fz1-GFP, which was detected in axons stained with phosphorylated MAP1B (MAP1BP). **(D) **Fz1-GFP clusters co-localize with pre-synaptic terminals loaded with FM4-64 under stimulation with 90 mM KCl (arrows). **(E) **Hippocampal neurons were transfected with Fz1-myc plus GFP to easily identify transfected neurons. Fz1 staining (red) is highly increased in transfected neurons (asterisk) compared to non-transfected neurons (arrow). **(F) **Bassoon puncta were analyzed in neurons transfected with Fz1-myc or with empty vector (pCS2+) using an anti-Bassoon antibody and quantifying the clusters per neurite length in GFP-positive axons. There was an increased number of Bassoon puncta in neurons transfected with Fz1-myc compared to control. *P < 0.05; **P < 0.01; NS, not significant. Error bars indicate standard error of the mean.

In order to determine whether Fz1 could have a presynaptic role, we evaluated Bassoon clustering during the initial stages of synaptic assembly in neurons treated for 24 h with different concentrations of the soluble Fz1 CRD (Figure 4A). In these hippocampal neurons at 9 DIV, there was a significant decrease in the density of Bassoon clusters after treatment with 1 μg/ml Fz1-CRD (34.42 ± 0.98 Bassoon puncta per 100 μm neurite length) compared to control (40.12 ± 1.07). This decrease was not observed when neurons were treated with 1 μg/ml of the soluble CRD of the Fz2 receptor, which has been shown to be involved in non-canonical *Wnt *pathways [22,23] (40.18 ± 0.73 Bassoon puncta per 100 μm), indicating a specific effect of Fz1 on Bassoon clustering that is not mediated by all Fz receptors. Figure 4B shows representative images of control neurons and neurons treated with 1 μg/ml Fz1-CRD or Fz2-CRD. This result suggests that Fz1 may normally participate in the presynaptic differentiation. To further assess this hypothesis, we overexpressed Fz1 in hippocampal neurons. First, to evaluate whether overexpressed Fz1 localizes at presynaptic sites, neurons were transfected with GFP-tagged Fz1. We observed that this receptor was present in axons stained with phosphorylated MAP1B (MAP1BP) antibody (Figure 4C, upper panel), showing a punctuate distribution at higher magnification (Figure 4C, lower panels). In addition, Fz1-GFP co-localized with active synaptic terminals that were loaded with FM4-64 under stimulation with KCl (Figure 4D, arrows), indicating a synaptic location of the overexpressed Fz1 receptor. To assess the effect of this overexpression on presynaptic differentiation, we transfected neurons with rat Fz1-myc, which we previously demonstrated to be able to increase the activation of the *Wnt *signaling pathway, indicating that this is a functional receptor [24]. Neurons were co-transfected with GFP to easily identify transfected neurons (Figure 4E, F). As determined by anti-Fz1 immunoreactivity (Figure 4E), Fz1 is highly overexpressed in transfected neurons (asterisk) compared to non transfected neurons (arrow), and this overexpressed receptor is located in axons stained with MAP1BP. We then evaluated Bassoon clustering during the initial stages of synaptic assembly. At 9 DIV, hippocampal neurons transfected with Fz1-myc showed increased numbers of Bassoon puncta compared to control neurons transfected with the empty vector (pCS2+; Figure 4F), strongly supporting a role for Fz1 in presynaptic differentiation.

### *Wnt-3a*/Fz1 signaling modulates presynaptic protein clustering and synaptic vesicle recycling

**Figure 5:**
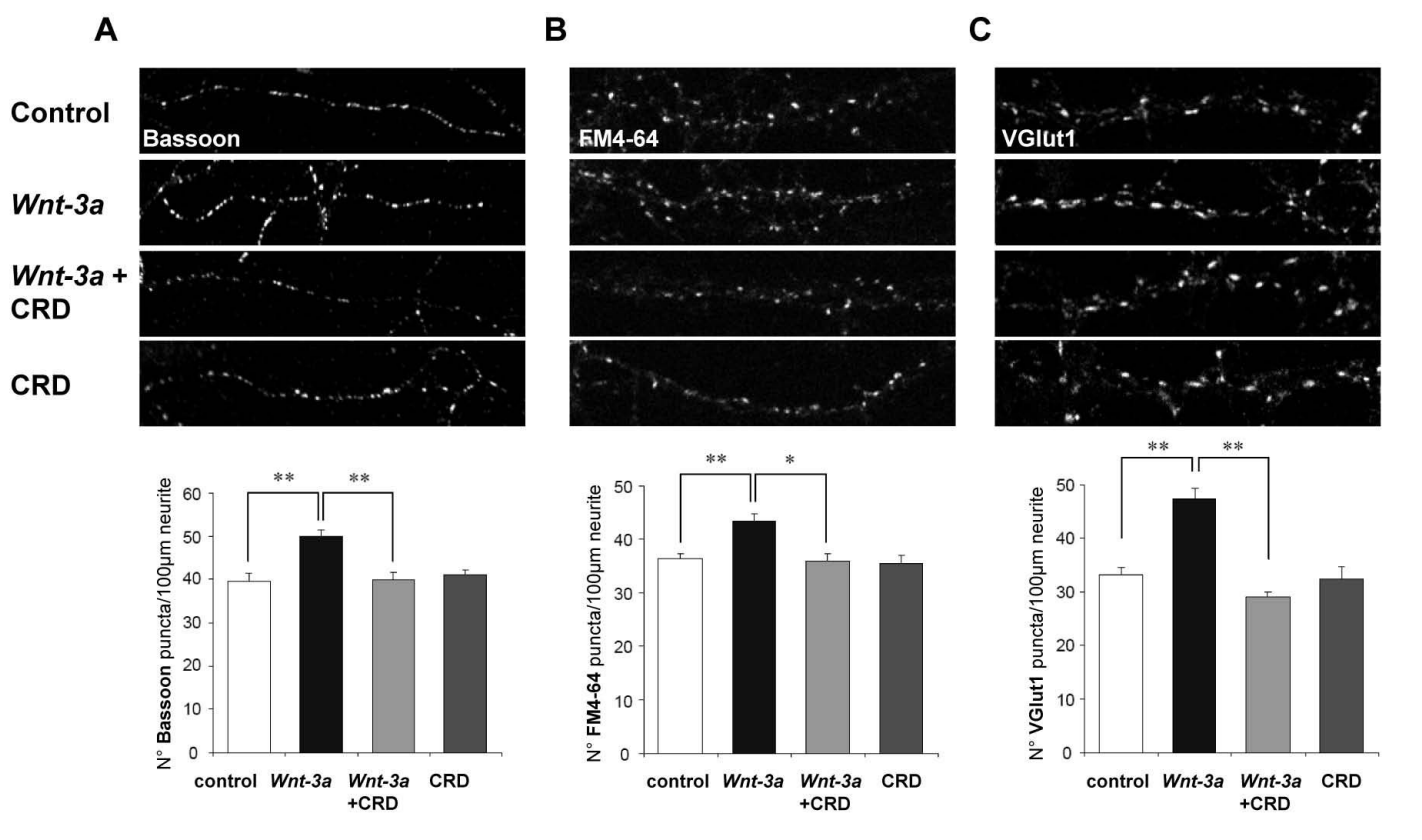
***Wnt-3a *induces presynaptic differentiation in cultured hippocampal neurons mediated by the Fz1 receptor**. **(A) **Neurons at 9 DIV exposed to *Wnt-3a *for 1 h show elevated numbers of Bassoon clusters detected by immunofluorescence. Addition of the soluble Fz1-CRD prevents this induction. **(B) **Synaptic vesicle recycling is visualized by FM4-64 uptake (11 DIV). One-hour treatment with *Wnt-3a *induced a significant increase in the number of FM4-64-positive sites, which was prevented by co-treatment with the Fz1-CRD. **(C) ***Wnt-3a *induces the number of VGlut1 clusters in neurons at 14 DIV, which was prevented by co-treatment with Fz1-CRD. Error bars indicate standard error of the mean. * *P *< 0.05; ** *P *< 0.01.

To better establish the role of Fz1 in synapse assembly, we evaluated the effect of the canonical *Wnt *ligand *Wnt-3a*. It has been previously shown that Fz1 is a functional partner for *Wnt-3a*, inducing TCF-dependent transcription [16]. In fact, we have demonstrated that *Wnt-3a *signaling is mediated by Fz1 in PC12 cells and, moreover, Fz1 modulates the protective effect of *Wnt-3a *against Aβ oligomers in hippocampal neurons [24]. So, in an attempt to understand the potential role of Fz1 in the synapse, we studied the effect of *Wnt-3a *on synaptic protein clustering. Treatment of hippocampal neurons at 10 DIV with 150 ng/ml *Wnt-3a *for 1 h induced an increase in the number of Bassoon clusters (50.04 ± 1.42 puncta per 100 μm) compared to control (39.68 ± 1.72 puncta per 100 μm) (Figure 5A). Interestingly, this effect was blocked by the soluble Fz1-CRD (39.92 ± 1.67 puncta per 100 μm; Figure 5A, graph), suggesting that this effect is mediated by the Fz1 receptor. Moreover, the number of functional, activity-dependent recycling presynaptic sites was also increased by *Wnt-3a *compared to control (Figure 5B), in agreement with our previous findings [13]. This effect was also blocked by Fz1-CRD, suggesting that it is mediated by Fz1. These results indicate that *Wnt-3a *regulates the formation of functional presynaptic terminals. The effect of *Wnt-3a *was also evaluated in older neurons (14 DIV) where treatment with *Wnt-3a *induced the clustering of VGlut1 (47.36 ± 1.88 puncta per 100 μm) compared to untreated neurons (36.38 ± 1.22 puncta per 100 μm) and to neurons treated with *Wnt-3a *plus Fz1-CRD (29.00 ± 0.89 puncta per 100 μm) (Figure 5C). At 14 DIV, *Wnt-3a *treatment also induced the clustering of SYP (control, 38.56 ± 2.04; *Wnt-3a*, 50.70 ± 2.71 puncta per 100 μm; *P *< 0.01). Considering the increase observed in presynaptic protein puncta by *Wnt-3a *treatment, we evaluated whether synaptic contact numbers, identified by PSD-95/Syn or PSD-95/SV2 co-clusters, were also affected in 14 DIV neurons. After a 1-h treatment, *Wnt-3a *did not increase the number of synapses per 100 μm neurite length (PSD-95/Syn_control_, 19.94 ± 2.37; PSD-95/Syn_*Wnt-3a*_, 19.32 ± 2.33; PSD-95/SV2_control_, 19.69 ± 3.23; PSD-95/SV2_*Wnt-3a*_, 20.96 ± 3.04). These results suggest that *Wnt-3a*/Fz1 signaling induces a fast increase in pre-synaptic protein clustering, which may precede the increase in synaptic contact number. Indeed, after 24 h of treatment, *Wnt-3a *induced an almost 20% increase in synaptic contact number, which was not observed in neurons treated with *Wnt-3a *plus Fz1-CRD (*Wnt-3a *versus control, 1.19 ± 0.05, *P *< 0.05; *Wnt-3a*+CRD versus control, 0.95 ± 0.04).

**Figure 6:**
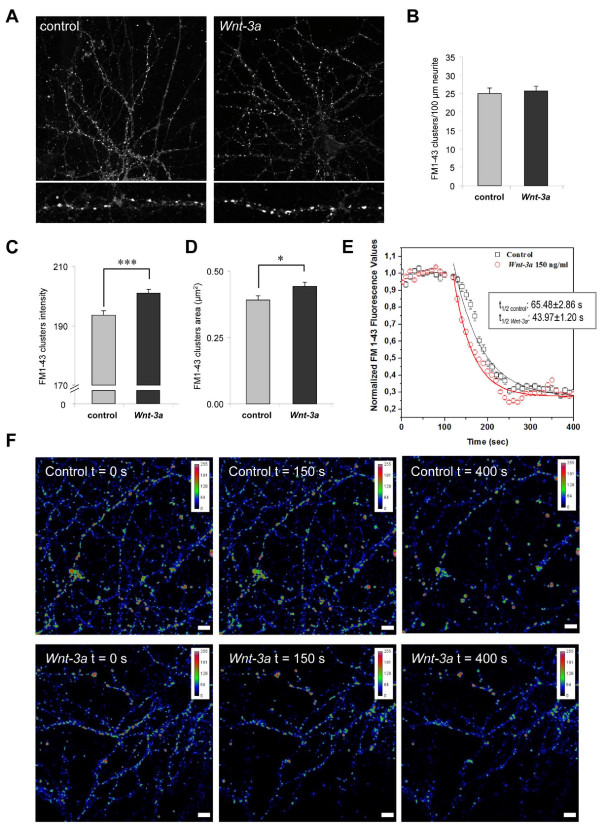
***Wnt-3a *stimulates synaptic vesicle release in mature cultured hippocampal neurons**. **(A) **Hippocampal neurons at 21 DIV were treated with control or *Wnt-3a*-conditioned media for 1 h and the efficiency of activity-dependent vesicle recycling was evaluated by measuring the uptake of the amphiphatic fluorescent dye FM1-43 in response to depolarization with 90 mM KCl. **(B-D) **Quantification of the number of FM1-43 per neurite length (B), the mean intensity of FM1-43 puncta (C) and the mean area of FM1-43 clusters (D). **(E, F) **After treatment with recombinant *Wnt-3a *or vehicle for 3 h, synaptic vesicles were loaded with FM1-43 by depolarization and the decrease in fluorescence intensity induced by 90 mM KCl was measured by confocal time lapse microscopy imaging. (E) Graph of the normalized FM1-43 fluorescence decrease in control and *Wnt-3a *treated neurons shows that the half-time (t_1/2_) of fluorescence decay was higher in the control than in *Wnt-3a*-treated neurons, indicating that *Wnt-3a *increased the rate of FM1-43 unloading. Data represent at least 100 sites of vesicle release. (F) pseudocolored images of FM1-43 fluorescence of representative synaptic vesicle exocytosis in control and *Wnt-3a *treated neurons at different times. The pseudocolor scale is shown at the top right of images. Error bars indicate standard error of the mean. * *P *< 0.05; *** *P *< 0.001.

The rate and efficacy of synaptic vesicle recycling at pre-synaptic nerve terminals determine normal synaptic transmission and play a role in several forms of synaptic plasticity [25]. Recently, we demonstrated that *Wnt-7a *modulates the synaptic vesicle cycle in presynaptic nerve terminals of hippocampal neurons and that this canonical *Wnt *ligand is able to increase the synaptic transmission in CA3-CA1 synapses of hippocampal slices by a presynaptic mechanism [13]. To evaluate if the Fz1 ligand *Wnt-3a *is able to modulate the pre-synaptic function, mature hippocampal neurons at 21 DIV were treated with control or *Wnt-3a *conditioned media for 1 h and the efficiency of the activity-dependent vesicle recycling was evaluated by measuring the uptake of the amphiphatic fluorescent dye FM1-43 in response to depolarization with 90 mM KCl (Figure 6A). In contrast to the effect observed in young neurons, no differences were found in the density of FM1-43 clusters in response to *Wnt-3a *in mature neurons at 21 DIV (Figure 6B), although the mean intensity and area of FM1-43 puncta were increased in *Wnt-3a*-treated neurons compared to control cells (Mean intensity_control_, 193.580 ± 1.55; Mean intensity_*Wnt-3a*_, 200.939 ± 1.24; Area_control_, 0.39 ± 0.02; Area_*Wnt-3a*_, 0.44 ± 0.02; Figure 6C and 6D). The fluorescence intensity of buttons depends on the number of labeled vesicles retrieved during endocytosis, and is indicative of the total functional recycling pool size of synaptic vesicles [26].

We also analyzed the probability of synaptic vesicle release. After treatment with recombinant *Wnt-3a *or vehicle for 3 h, synaptic vesicles were loaded with FM1-43 by depolarization with 60 mM KCl and the decrease in fluorescence intensity induced by the activity dependent exocytosis at 90 mM KCl was measured by confocal time lapse microscopy imaging. As indicated in Figure 6E, F, pre-incubation of neurons with *Wnt-3a *increased the rate of release of the FM1-43 fluorescence trapped in vesicles. The half-time (t_1/2_) of fluorescence decay in *Wnt-3a*-treated neurons was 43.97 ± 1.20 s versus 65.48 ± 2.86 s for control neurons, indicating that *Wnt-3a *increased the rate of FM1-43 unloading. The effect of *Wnt-3a *was analyzed in a total of more than 100 sites of vesicle release. These results indicate that a *Wnt-3a*-dependent signaling modulates the activity of the presynaptic compartment, increasing the rate of release of synaptic vesicles.

## Discussion

The *Wnt *signaling pathway regulates several fundamental developmental processes and modulates synaptic structure and function. Although roles for *Wnt *ligands in regulating synaptic assembly and plasticity have been shown [10,13,14], little is known about the receptors that mediate these effects. Previously, we and others determined that Fz1 is highly expressed in the hippocampus [24,27,28]. In the present work, we determined the synaptic distribution of the canonical *Wnt *receptor Fz1. We found that this receptor is located at synaptic sites in hippocampal neurons, co-localizing with presynaptic proteins and with active synaptic vesicle recycling sites stained under KCl stimulation with a fluorescent dye, indicating that this receptor is distributed presynaptically. In addition, Fz1 was detected in a synaptosome preparation from adult rat brains, supporting the synaptic distribution of this receptor.

Specifically, the presynaptic distribution of Fz1 determined in this work suggests that the activation of the *Wnt *signaling pathway through Fz1 could be operating at the presynaptic level, where it could modulate presynaptic structure and function. Consistent with this view, we observed that overexpression of Fz1 induced the clustering of Bassoon, which is a component of the presynaptic cytoskeletal matrix involved in the structural organization of neurotransmitter release sites and is delivered to nascent synapses via vesicles that are detectable early during the formation of synaptic junctions [21,29]. Furthermore, treatment with the CRD of Fz1, the extracellular region of the receptor that binds *Wnt *molecules with high affinity [3], decreased the number of Bassoon puncta per neurite length, indicating that activation of endogenous Fz1-mediated signaling contributes to synapse formation. This effect was not observed when neurons were treated with the CRD of the Fz2 receptor, which has been shown to activate non-canonical *Wnt *pathways [22,23]. The specificity of the *Wnt*-Fz interaction remains largely unknown, particularly in vertebrates, because of the large number of *Wnts *and Fzs; however, it is well known that *Wnt-3a *and Fz1 are functional partners [16,24,30]. We observed that *Wnt-3a *induced the clustering of Bassoon during the initial stages of synaptic assembly, increased synaptic vesicle recycling and increased the clustering of VGlut1. All these effects were prevented by co-treatment with the CRD of Fz1, suggesting the involvement of *Wnt-3a*/Fz1 signaling. VGluts are vesicular glutamate transporters that mediate the transport of glutamate from the cytoplasm into synaptic vesicles; they are therefore used as specific markers of the glutamatergic phenotype [31]. VGlut1 is the major isoform in cortex, hippocampus and cerebellar cortex. The increase in the number of VGlut1 clusters indicates that *Wnt-3a*/Fz1 signaling increased the number of excitatory presynaptic puncta. In addition, we observed an increase in the number of SYP clusters, a synaptic vesicle membrane protein that has been associated with synaptic vesicle cycling and was shown to regulate activity-dependent synapse formation [32]. Altogether, the increases in vesicle-associated and active zone protein clusters suggest that the *Wnt-3a*/Fz1 pathway modulates the assembly of presynaptic terminals. These pre-synaptic effects were observed after 1 h of treatment, whereas no effect on synaptic contact number was observed. On the other hand, in 24-h experiments, *Wnt-3a *did induce an increase in synaptic contact number that was prevented by co-treatment with the CRD of Fz1. These results suggest that *Wnt-3a*/Fz1 signaling may induce a fast increase in pre-synaptic protein clustering that may precede the increase in synaptic contacts.

Synaptic vesicles accumulate neurotransmitters and secrete them upon stimulation. The recycling process of synaptic vesicles, including exocytosis, endocytosis and re-exocytosis, is essential for maintaining vesicle pools in the nerve terminals and ensuring normal synaptic transmission. The rate of vesicle turnover and the size of vesicle pools play a role in several forms of synaptic plasticity [25]. Recently, we demonstrated that the canonical *Wnt-7a *ligand modulates the recycling and release of synaptic vesicles in functionally mature excitatory synapses *in vitro*. Here, we determined that *Wnt-3a *treatment increased the number of functional presynaptic sites in mature neurons, which is in agreement with previous findings showing that *Wnt-3a *increases the number of active recycling sites [13]. Moreover, *Wnt-3a *increased the efficacy of synaptic vesicle exocytosis in synaptically mature neurons. These results suggest a role for the *Wnt-3a*/Fz1 pathway in presynaptic function in adult neurons. Interestingly, we have observed strong staining for synaptic Fz1 in older neurons (21 to 28 DIV), and the expression of Fz1 in the hippocampus increases gradually until adult stages, supporting the idea that it may have a role in mature synaptic function.

Thus, our results indicate that *Wnt-3a*/Fz1 signaling modulates the structure and function of the presynaptic compartment. This modulation is probably mediated by the activation of components of the canonical *Wnt *pathway, since Fz1 and *Wnt-3a *have been widely associated with this branch of *Wnt *signaling [16,24,33]. In agreement with this idea, treatment with Dickkopf-1, which promotes internalization of the LRP5/6 co-receptor, required for canonical signal activation but not for non-canonical *Wnt *signaling, resulted in decreased numbers of excitatory presynaptic puncta, indicating that activation of the endogenous canonical pathway contributes to synapse formation [11]. In addition, it has been recently shown that Wingless (the *Drosophila Wnt *homolog) directly signals to the presynaptic endings at the *Drosophila *neuromuscular junction, where it activates components of the canonical pathway and locally regulates microtubules [34].

It has been previously suggested that activation of the canonical and non-canonical pathways differentially modulates pre- and postsynaptic events. The non-canonical ligand *Wnt-5a *induces the clustering of the postsynaptic protein PSD-95 and glutamate receptors [35,36] and *Wnt-7b *increases dendritic branching in cultured hippocampal neurons through Rac and JNK [9], whereas the canonical *Wnt-7a *ligand induces presynaptic protein clustering [8,11] and the recycling and exocytosis of synaptic vesicles in cultured hippocampal neurons, and enhances synaptic transmission in adult hippocampal slices [13]. Interestingly, loss of function of the canonical Wnt pathway in the presynaptic region, but not in the postsynaptic muscles of the *Drosophila *neuromuscular junction, affects synaptic differentiation [34]. This is in agreement with our findings, which suggest a presynaptic effect of canonical Fz1/Wnt-3a signaling. It will be interesting to study whether all the synaptic effects of Wnts are modulated by specific receptors whose expression and localization in neurons should be correlated with their functions. We have determined that Fz receptors have varied distributions in neurons and show very different patterns of expression in the developing hippocampus (unpublished data). Thus, the activation of specific *Wnt *signaling pathways would be controlled temporally and spatially during the development of neuronal circuits. In addition, there are alternative *Wnt *receptors [37] that could spatially modulate the activation of the *Wnt *pathway. This is the case for the axonal localization of Ryk receptors in mammals and *Drosophila *[38], and the Ror2 receptor [39], which is highly concentrated in the growth cones of immature neurons and are present throughout the somatodendritic compartment of mature hippocampal cells [40].

In summary, we show for the first time the presynaptic distribution of a Fz receptor in mammalian neurons, which could mediate the synaptic effects of the *Wnt *signaling pathway activation. This synaptic localization suggests that there could be a local activation of the *Wnt *pathway at the synapse. In addition, the synaptic expression of some downstream components of the *Wnt *pathway have been described [10,11,14,34,41], indicating that the machinery required for the local activation of the pathway is present at central synapses. Altogether, these results suggest that *Wnts *binding to synaptic Fz-LRP5/6 could activate the canonical *Wnt *signaling at the synapse and, as a consequence, increase presynaptic inputs and the recycling and release of synaptic vesicles. Our findings give new insight into the mechanisms by which the *Wnt *signaling pathway could modulate the synapse.

## Materials and methods

### Primary culture of rat hippocampal neurons

Rat hippocampal cultures were prepared as described previously [42,43]. Hippocampi from Sprague-Dawley rats at embryonic day 18 were removed, dissected free of meninges in Ca^2+^/Mg^2+^-free Hanks' balanced salt solution (HBSS), and rinsed twice with HBSS by allowing the tissue to settle to the bottom of the tube. After the second wash, the tissue was resuspended in HBSS containing 0.25% (w/v) trypsin and incubated for 15 minutes at 37°C. After three rinses with HBSS, the tissue was mechanically dissociated in plating medium (Dulbecco's modified Eagle's medium (GIBCO, Rockville, MD, USA)), supplemented with 10% horse serum (GIBCO), 100 U/ml penicillin, and 100 μg/ml streptomycin by gentle passage through Pasteur pipettes. Dissociated hippocampal cells were seeded onto poly-L-lysine-coated six-well culture plates at a density of 7 × 10^5 ^cells per well in plating medium. Cultures were maintained at 37°C in 5% CO_2 _for 2 h before the plating medium was replaced with neurobasal growth medium (GIBCO) supplemented with B27 (GIBCO), 2 mM L-glutamine, 100 U/ml penicillin, and 100 μg/ml streptomycin. On day 2, cultured neurons were treated with 2 μM cytosine arabinoside (AraC) for 24 h; this method resulted in cultures highly enriched for neurons (approximately 5% glia). For *Wnt-3a *treatments, neurons were treated with 150 ng/ml recombinant *Wnt-3a *(R&D Systems, Minneapolis, MN, USA) and with 300 ng/ml recombinant Fz-1-CRD/Fc Chimera (R&D Systems).

### Immunofluorescence

Hippocampal neurons were seeded onto poly-L-lysine-coated coverslips in 24-well culture plates at a density of 2.5 × 10^4 ^cells per well. Cells were rinsed twice in ice-cold phosphate-buffered saline (PBS) and fixed with a freshly prepared solution of 4% paraformaldehyde in PBS for 20 minutes and permeabilized for 5 minutes with 0.2% Triton X-100 in PBS. After several rinses in ice-cold PBS, cells were incubated in 0.2% gelatin in PBS (blocking solution) for 30 minutes at room temperature, followed by an overnight incubation at 4°C with primary antibodies. Cells were extensively washed with PBS and then incubated with Alexa-conjugated secondary antibodies (Molecular Probes, Carlsbad, CA, USA) for 30 minutes at 37°C. Coverslips were mounted in mounting medium and analyzed on a Zeiss LSM 5 Pascal confocal microscope. Primary antibodies used were goat anti-Fz1 (R&D Systems), rabbit anti-Synapsin I (Santa Cruz Biotechnology Inc., Santa Cruz, CA, USA), rabbit anti-VAMP (Santa Cruz Biotechnology Inc.), goat anti-Synapsin I (Santa Cruz Biotechnology Inc.), goat anti-SYP (Santa Cruz Biotechnology Inc.), monoclonal anti-Bassoon antibody (Assay designs, Ann Arbor, MI, USA), and monoclonal anti-MAP1BP antibody (Sternberger Monoclonals, Baltimore, MD, USABaltimore, MDBaltimore). The monoclonal antibodies anti-PSD-95 and anti-VGlut1 were developed by and obtained from the UC Davis/NIH NeuroMab Facility, supported by NIH grant U24NS050606 and maintained by the Department of Neurobiology, Physiology and Behavior, College of Biological Sciences, University of California, Davis, CA, USA.

Images were captured with a Zeiss LSM 5 Pascal confocal microscope. Images were analyzed using NIH ImageJ software. Co-localization analysis and quantification of the number of puncta were carried out under threshold conditions to identify independent clusters. Co-localization analysis was performed on randomly selected images, using the NIH ImageJ software with the co-localization analysis plug-in. Mander's coefficients represent the number of co-localized pixels [17]; they range from 0 to 1, indicating no co-localization to complete co-localization, and are independent of the pixel intensities within each respective channel.

### FM uptake

FM4-64 FX or FM1-43 (Molecular Probes) were added at a concentration of 15 μM to Tyrode saline solution (119 mM NaCl, 2.5 mM KCl, 2 mM CaCl_2_, 2 mM, MgCl_2_, 25 mM HEPES, 30 mM glucose buffered to pH 7.4). Neurons were incubated with this dye solution for 1 minute and then the FM dye was loaded using 90 mM KCl stimulation for 30 s. Neurons were washed five times for 10 minutes in dye-free Tyrode solution to decrease background staining of the membrane. For some of the experiments, neurons were fixed in 4% paraformaldehyde in PBS for 20 minutes and FM4-64 puncta were analyzed by confocal microscopy. For retrospective immunofluorescence the neurons on coverslips were mounted in a microscope perfusion chamber and stained with FM1-43 as described above. Images were taken before and after neurons were destained in high KCl solution for 30 s. Cells were then fixed and permeabilized with 0.2% Triton X-100 for 10 minutes to wash out the FM4-64 fluorescence and processed for immunofluorescence.

### Synaptosome preparation

Synaptosomes were isolated from adult rat brain using the Percoll gradient method [44]. In brief, adult mouse brain was homogenized in buffer A (0.32 M sucrose, 5 mM Hepes, 0.1 mM EDTA, pH 7.4, plus protease and phosphatase inhibitors) at 800 rpm ten times at 4°C, and then centrifuged at 1,000 × *g *for 10 minutes. The supernatant was centrifuged at 10,000 × *g *for 25 minutes, and the pellet was resuspended in buffer B (0.25 M sucrose, 5 mM Hepes, 0.1 mM EDTA, pH 7.5) 8.5% Percoll, and layered on top of a Percoll discontinuous gradient. Synaptosomes were taken from a 10 to 16% interface and washed in buffer B. Proteins were quantified using the BCA protein assay kit (Pierce, Rockford, IL, USA) and analyzed by immunoblotting. The relative purity of the synaptosome preparations was established by electron microscopy.

### Immunoblot analysis

Neurons growing on six-well culture plates or hippocampus obtained from rat brains at different ages were lysed in ice-cold lysis buffer (10 mM Tris-HCl, pH 7.8, 100 mM NaCl, 10 mM EDTA, 0.5% Nonidet P-40, and 0.5% sodium deoxycholate) supplemented with protease inhibitors. The homogenates were maintained in ice for 30 minutes and then neuronal culture homogenates were centrifuged at 1,000 × *g *for 5 minutes (4°C) and hippocampus homogenates were centrifuged at 15,000 × *g *for 10 minutes (4°C) to remove nuclei and large debris. The supernatant was recovered and protein concentration was determined by BCA protein assay kit (Pierce). Proteins were resolved in SDS-PAGE (10% polyacrylamide), transferred to PVDF membrane and reacted with primary antibodies. The reactions were followed by incubation with peroxidase-labeled secondary antibodies (Pierce) and developed using the ECL technique (PerkinElmer, Waltham, MA, USA). Primary antibodies were the same used for immunofluorescence in addition to rabbit anti-β-tubulin (Santa Cruz Biotechnology Inc.) and rabbit anti-N-cadherin (Santa Cruz Biotechnology Inc.).

### Neuronal transfection

Neurons were transfected using LipofectAMINE 2000 (Invitrogen, Carlsbad, CA, USA) 2 days after seeded on cover slips in 24-well culture plates at a density of 4 × 10^4 ^cells per well. Briefly, 0.25 μg of Fz1-GFP plasmid or GFP vector and 0.75 μl of LipofectAMINE 2000 were mixed in 100 μl of OptiMEM (GIBCO) according to the manufacturer's instructions. For the co-transfection of Fz1-Myc with GFP the amounts were 0.35 μg and 0.15 μg, respectively. After 20 minutes the DNA-LipofectAMINE 2000 Reagent complex was added to the cells. Neurons were incubated for 2 h at 37°C and then the media was replaced with Neurobasal growth medium (GIBCO) supplemented with B27 (GIBCO), 2 mM L-glutamine, 100 U/ml penicillin, and 100 μg/ml streptomycin.

### Imaging of FM1-43 destaining in presynaptic terminals of cultured hippocampal neurons

Hippocampal neurons at 21 DIV were incubated for 3 h with *Wnt-3a *(150 ng/ml) or vehicle at 37°C. Neurons on coverslips were then washed with Tyrode modified solution, mounted in a microscope perfusion chamber, and incubated for 30 s with 10 μM FM1-43 (Molecular Probes) followed by 1 minute of loading by mild depolarization with 30 mM KCl. Nonspecific and non-synaptic FM1-43 staining was diminished by washing with 10 minutes of continued perfusion of Tyrode solution at 1 to 2 ml/minute controlled with a peristaltic pump (Cole Palmer, Vernon Hills, IL, USA). The chamber was adapted at the stage of a Zeiss Axiovert 200 M microscope coupled to Pascal LSM5 confocal laser scanning system. Neurons were imaged with a 63 × 1.4 NA oil objective at 512 × 512 full-frame resolution using a 488-nm argon laser to excite the FM1-43 probe, and the fluorescence signals were collected over 505 nm. Then, after a period of 50 s of basal fluorescence acquisition, neurons were depolarized with 90 mM KCl and imaged for 300 s at 1-s intervals. Images from presynaptic loaded puncta were selected for measuring fluorescence intensities using areas of the region of interest of 1.5 × 1.5 μm. Images of *Wnt-3a*-treated neurons and control neurons were obtained using identical settings for laser power, confocal thickness, and detector sensitivity. All measurements were taken at room temperature (25°C).

### Statistical analysis

Statistical analysis was performed using statistical software Prism 5 (GraphPad Software Inc., San Diego, CA, USA). Values are expressed as mean ± standard error of the mean. Statistical significance of differences was assessed with the non-paired Student's *t*-test or ANOVA, and non-normally distributed data were analyzed using the Mann-Whitney test or Kruskal Wallis (*P *< 0.05 was considered significant).

## Abbreviations

CRD: cysteine-rich domain; DIV: days *in vitro*; Fz: Frizzled; GFP: green fluorescent protein; HBSS: Hanks' balanced salt solution; MAP1BP: phosphorylated MAP1B; PBS: phosphate-buffered saline; Syn: synapsin I; SYP: synaptophysin; VGlut1: vesicular glutamate transporter 1.

## Competing interests

The authors declare that they have no competing interests.

## Authors' contributions

LV-N performed the experiments on the expression and distribution of Fz1 in neurons and the effects of *Wnt-3a *and Fz1 on presynaptic differentiation, and drafted the manuscript. CPG carried out the co-localization analysis of Fz1 in functional synapses and the preparation of synaptosomal fractions. IEA carried out the imaging of FM1-43. ARA participated in the design of experiments. NCI participated in the design of the experiments and writing of the manuscript. All authors read and approved the final manuscript.
